# Pathogenesis of Bone Diseases: The Role of Immune System

**DOI:** 10.1155/2015/785845

**Published:** 2015-05-04

**Authors:** Giacomina Brunetti, Giorgio Mori, Patrizia D'Amelio, Roberta Faccio

**Affiliations:** ^1^Section of Human Anatomy and Histology, Department of Basic Medical Sciences, Neuroscience and Sense Organs, University of Bari, 70124 Bari, Italy; ^2^Department of Clinical and Experimental Medicine, University of Foggia, 71100 Foggia, Italy; ^3^Section of Gerontology and Bone Metabolism Diseases, Department of Medical Science, University of Torino, 10126 Torino, Italy; ^4^Department of Orthopedics, Washington University School of Medicine, St. Louis, MO 63110, USA

Bone is a metabolically active tissue that undergoes continuous remodeling by two sequential events, bone formation and resorption. These events are strongly linked and tightly regulated to maintain skeletal homeostasis. The bone cells responsible for the dual events include the bone-resorbing cells, the osteoclasts, arising from monocyte-macrophage precursors, and the bone forming cells, the osteoblasts, having a mesenchymal origin. Immune and bone cell activities are linked by several pathways [1] and the former can promote bone building or destruction. Further, immune cells can be involved in the mineralization process occurring in extra-skeletal sites. In this special issue different authors highlighted these items both through research articles and reviews.

In detail, interaction between osteoblast precursors, the mesenchymal stem cells (MSCs) [2], and immune cells during fracture repair acts as one of the key factors governing successful bone healing. Additionally, bone damage following immune deregulation may be local as in arthritis and periodontal disease (PD) or systemic as in osteoporosis [3] and osteotropic cancers [4, 5]. It could be multifactorial and thus due to genetic modifications (i.e., Gaucher disease) as well as to lipopolysaccharide- (LPS-) mediated release of inflammatory cytokines (i.e., PD, osteomyelitis, and arthritis), and so forth. New insights suggest that, in immune-mediated bone diseases, bone resorption active phases are characterized by increased levels of immunoreceptor tyrosine-based activation motifs (ITAMs); these molecules together with OSCAR could be indicative of disease progression. Further, osteotropic cancer-related immune alterations showed distinct immune cell phenotype as observed in chronic myeloid leukemia, multiple myeloma, and bone metastatic solid tumors.

State-of-the-art and new mechanisms are clearly described in this special issue; they can be useful for the identification of new therapeutic targets and bone disease markers.

*Giacomina Brunetti* *Giacomina Brunetti* *Giorgio Mori* *Giorgio Mori* *Patrizia D'Amelio* *Patrizia D'Amelio* *Roberta Faccio* *Roberta Faccio*
